# Supplementary figures and images for: Quasi-Continuous Network Structure Greatly Improved the Anti-Arc-Erosion Capability of Ag/Y2O3 Electrical Contacts
Source: Materials (Basel). 2022 Mar 26;15(7):2450. doi: 10.3390/ma15072450 (PMC8999512; doi:10.3390/ma15072450)

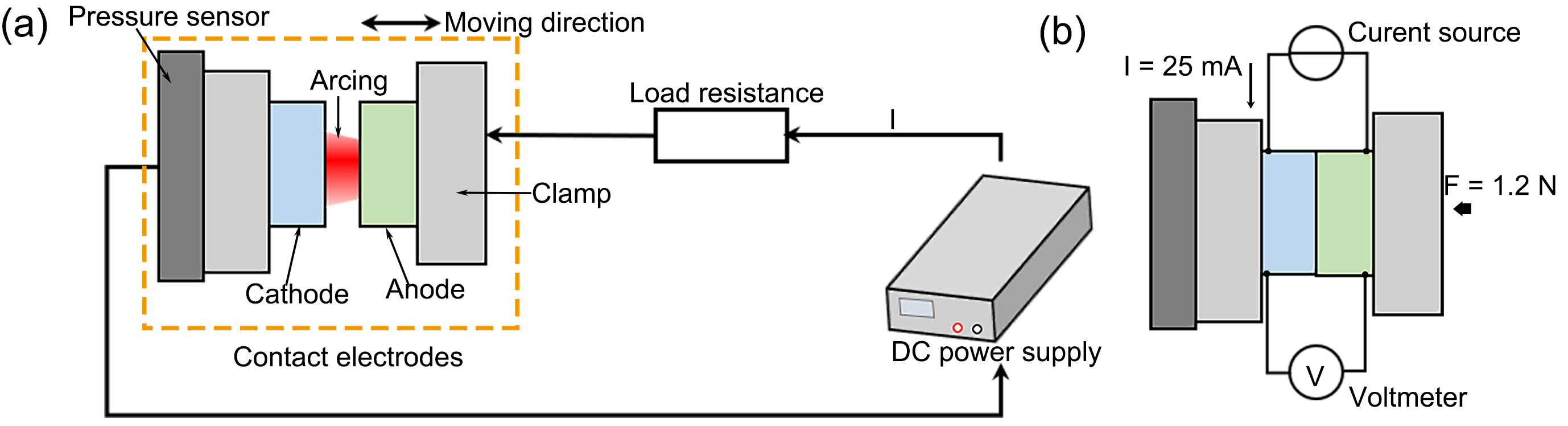

Supplement: Supplementary file 1 [file materials-15-02450-s001.zip › materials-1615701-supplementary/Figure S1.tif]

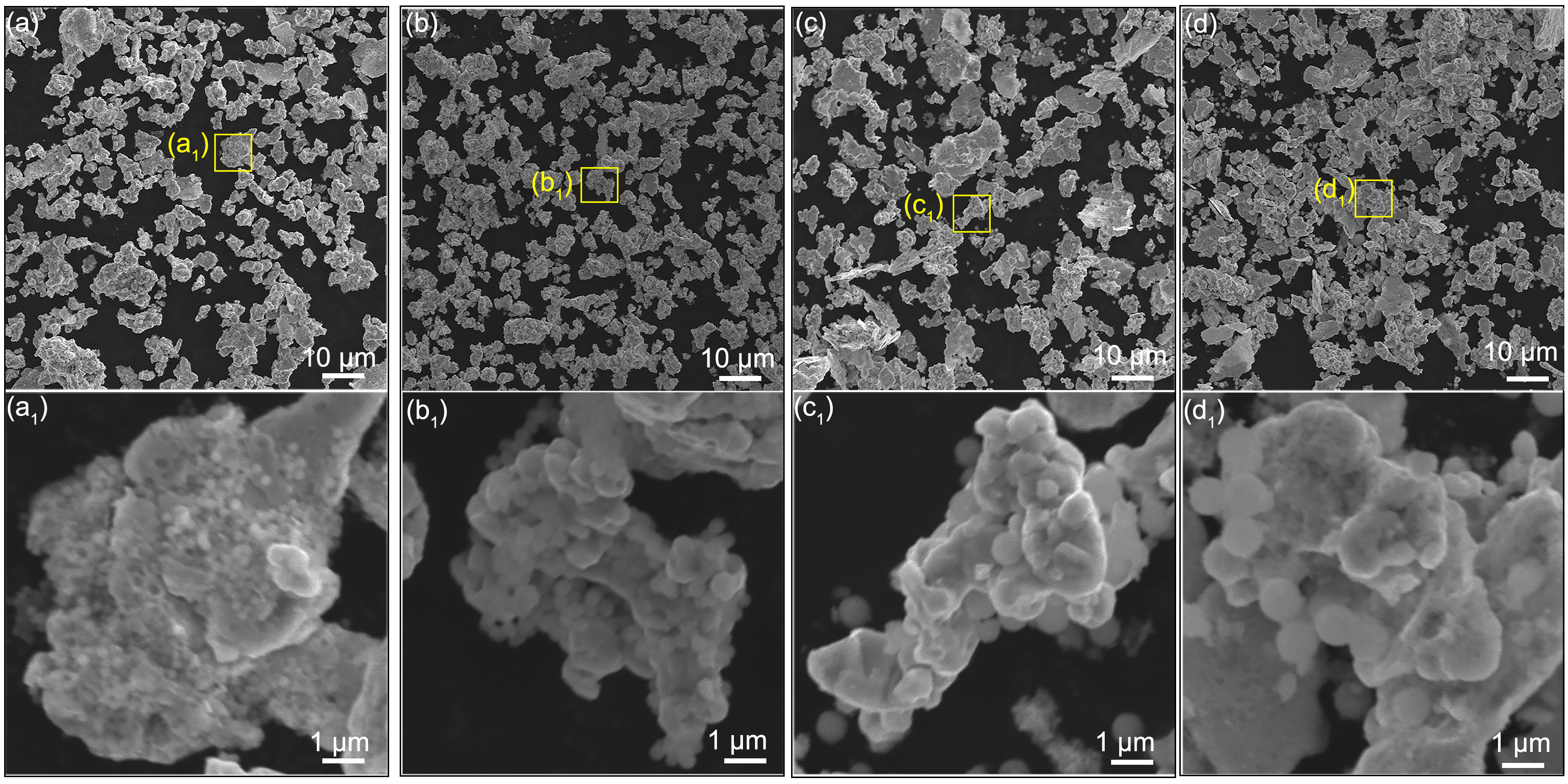

Supplement: Supplementary file 1 [file materials-15-02450-s001.zip › materials-1615701-supplementary/Figure S2.tif]

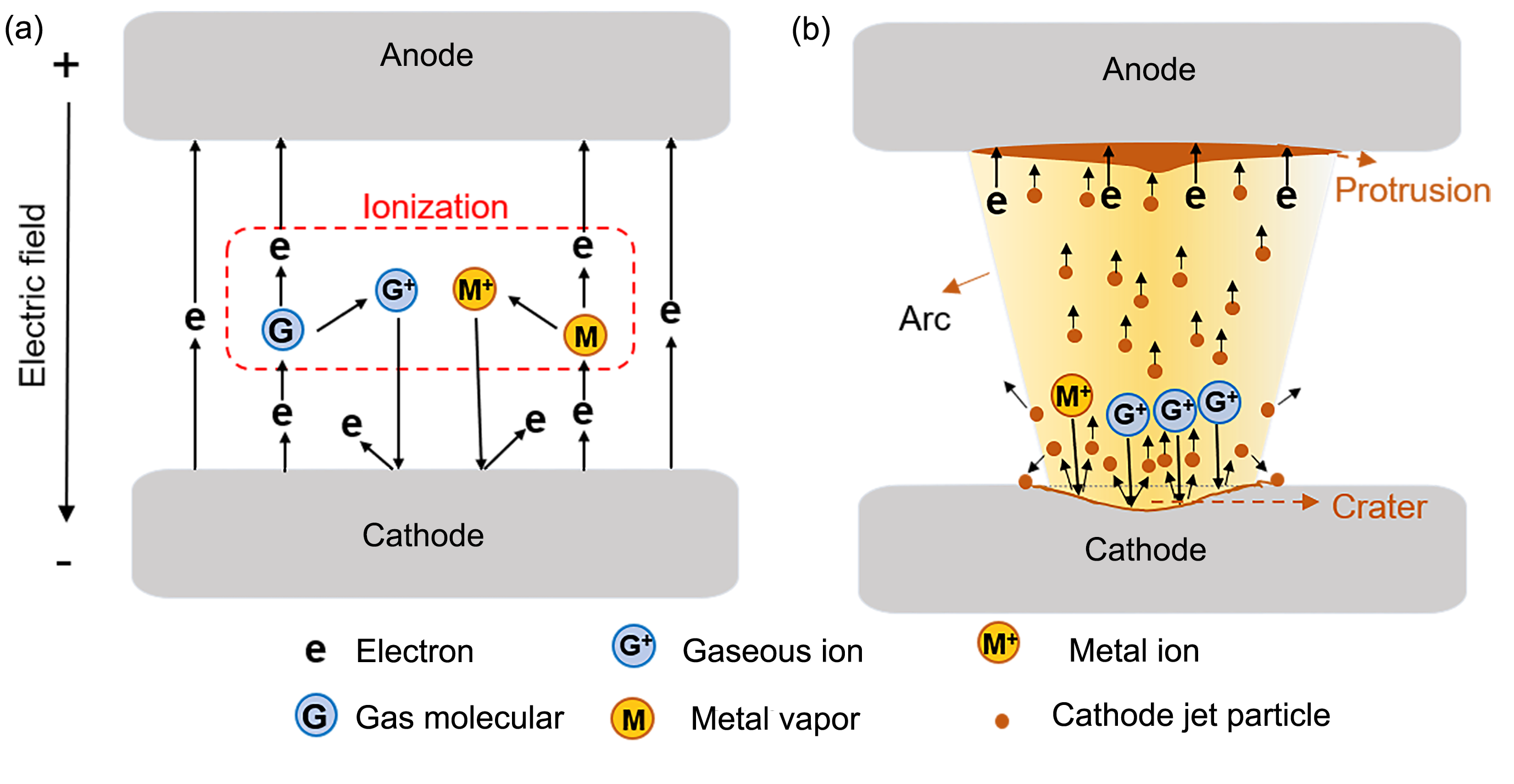

Supplement: Supplementary file 1 [file materials-15-02450-s001.zip › materials-1615701-supplementary/Figure S3.tif]
